# Process Development for Fabricating 3D-Printed Polycaprolactone-Infiltrated Hydroxyapatite Bone Graft Granules: Effects of Infiltrated Solution Concentration and Agitating Liquid

**DOI:** 10.3390/biomedicines12092161

**Published:** 2024-09-23

**Authors:** Faungchat Thammarakcharoen, Autcharaporn Srion, Waraporn Suvannapruk, Watchara Chokevivat, Wiroj Limtrakarn, Jintamai Suwanprateeb

**Affiliations:** 1 Biofunctional Materials and Devices Research Group, National Metal and Materials Technology Center (MTEC), National Science and Technology Development Agency (NSTDA), Khlong Nueng, Khlong Luang, Pathum Thani 12120, Thailand; faungcht@mtec.or.th (F.T.); autchars@mtec.or.th (A.S.); waraporn@mtec.or.th (W.S.); watcharc@mtec.or.th (W.C.); 2 Department of Mechanical Engineering, Faculty of Engineering, Thammasat University, Khlong Nueng, Khlong Luang, Pathum Thani 12120, Thailand; limwiroj@engr.tu.ac.th; 3 Thammasat University Center of Excellence in Computational Mechanics and Medical Engineering, Thammasat University, Khlong Nueng, Khlong Luang, Pathum Thani 12120, Thailand

**Keywords:** hydroxyapatite, polycaprolactone, composite, three dimensional printing, binder jetting

## Abstract

Bone grafts are commonly used in orthopedic and dental surgeries to facilitate bone repair and regeneration. A new type of bone graft, polycaprolactone-infiltrated three dimensionally printed hydroxyapatite (3DP HA/PCL), was previously developed by infiltrating polycaprolactone (PCL) into preformed three-dimensional-printed hydroxyapatite (3DP HA) that was fabricated using binder jetting technology combined with a low-temperature phase transformation process. However, when producing small granules, which are often used for bone grafting, issues of granule agglomeration emerged, complicating the application of this method. This study aimed to develop a fabrication process for 3DP HA/PCL bone graft granules using solution infiltration and liquid agitation. The effects of varying PCL solution concentrations (40% and 50% *w*/*w*) and different agitating liquids (deionized water or DI, N-Methyl-2-Pyrrolidone or NMP, and an NMP-DI mixture) on the properties of the resulting composites were investigated. XRD and FTIR analysis confirmed the coexistence of HA and PCL within the composites. The final PCL content was comparable across all conditions. The contact angles of 3DP HA/PCL were 26.3 and 69.8 degree for 40% and 50% PCL solution, respectively, when using DI, but were zero when using NMP and NMP-DI. The highest compression load resistance and diametral tensile strength were achieved using the 50% PCL solution with DI or the NMP-DI mixture. DI resulted in a dense PCL coating, while NMP and the NMP-DI mixture produced a porous and irregular surface morphology. All samples exhibited a porous internal microstructure due to PCL infiltration into the initial pores of the 3D-printed HA. Biocompatibility tests showed that all samples supported the proliferation of MC3T3-E1 cells, with the greatest OD values observed for the 50% PCL solution with DI or the NMP-DI mixture at each cultured period. Considering the microstructural, mechanical, and biological properties, the 50% PCL solution with the NMP-DI mixture demonstrated overall desirable properties.

## 1. Introduction

Bone grafting is essential in orthopedic and dental surgeries for bone repair and regeneration. Traditional bone graft materials like autografts, allografts, and xenografts have limitations, including donor site morbidity, limited supply, and disease transmission risks [1,2]. This has led to a growing interest in synthetic bone graft materials that can overcome these challenges. Among these, calcium phosphate-based materials, particularly hydroxyapatite (HA) and tricalcium phosphate (TCP), have garnered significant attention due to their excellent biocompatibility, osteoconductivity, and similarity to natural bone mineral [2,3]. Three-dimensional printing or additive manufacturing technology has introduced new possibilities for bone grafting and tissue engineering, enabling the production of bioceramic grafts and scaffolds with customized geometries and properties [4,5,6,7,8,9,10]. Various 3D printing methods, such as material extrusion, powder bed fusion, vat photopolymerization, material jetting, binder jetting, directed energy deposition, and sheet lamination, have been used in the fabrication of medical devices and tissue constructs [11,12,13,14]. Each technique offers unique benefits and drawbacks, making the selection of the most suitable method dependent on the specific requirements of the intended application and required properties.

Among the various 3D printing methods, binder jetting which involves selectively depositing a liquid binder onto a powder bed in a layer-by-layer fashion to construct a three-dimensional structure presents several potential advantages over other techniques. These advantages include the ability to utilize a wide range of powdered materials, processed at room temperature and ambient conditions, eliminating the need for support structures, and achieving a high fabrication speed [15,16]. However, binder jetting also has limitations, such as lower mechanical strength, the potential for part distortion during the densification process, high surface roughness, and reduced printing resolution. Despite these challenges, several studies have successfully employed binder jetting to create calcium phosphate scaffolds [17,18,19,20,21,22].Among several studies, it has been shown that binder jetting, using calcium sulfate-based powders and a water-based binder combined with low-temperature phase transformation, is an effective method for producing different forms of calcium phosphate, such as hydroxyapatite, brushite (dicalcium phosphate dihydrate), and monetite (dicalcium phosphate anhydrous) [23,24,25]. These 3DP calcium phosphates are notable for their high porosity, low crystallinity, resorbability, wicking ability, and bioactivity. They have been proven safe and effective for clinical applications in bone grafting, particularly in alveolar ridge preservation [26] and in creating customized bone block grafts tailored to specific anatomical needs [27]. Additionally, these grafts have demonstrated dual functionality, providing both drug delivery and antimicrobial properties, alongside bone regeneration capabilities in laboratory and clinical settings [28]. However, the mechanical properties of 3DP calcium phosphates produced by this technique are relatively low compared to high-temperature sintered calcium phosphate bone grafts, limiting their ability to withstand stress in certain applications. This limitation is due to their high porosity and the low compaction characteristic of the binder jetting process.

To enhance the mechanical properties of 3DP structures produced by binder jetting, infiltration techniques are often employed. These techniques introduce a reinforcing phase into the preformed structure, resulting in composites with improved mechanical properties. For 3DP calcium phosphate, particularly HA (3DP HA), various materials have been used for this purpose ranging from polymers to glass ceramic [29,30,31,32]. Recently, polycaprolactone (PCL) infiltrated 3DP HA composites (3DP HA/PCL) have been developed to enhance toughness and strength while maintaining biocompatibility and bioactivity [33]. PCL, a biodegradable polyester, was chosen as the infiltrant due to its favorable mechanical properties, degradability, and extensive use as an implant. PCL provides initial structural integrity and degrades over time to create a porous architecture conducive to tissue ingrowth.

In surgical procedures, bone grafts are used as granules or porous blocks [34]. Bone graft granules have advantages in terms of their versatility, ease of application, and ability to conform to irregularly shaped defects, and the resorption bone formation can occur throughout the defect, as it provides a balance between ease of handling, effective packing within bone defects, and suitable biological response. Granule size is a crucial factor in bone healing, primarily due to differences in the surface-to-volume ratio [35]. The size of granules are key in shaping the three-dimensional scaffold structure, which in turn influences bone formation. Smaller granules offer a higher surface area-to-volume ratio, which enhances osteoconductivity and allows for greater cell attachment and infiltration. However, their small size can also lead to faster resorption and may require additional structural support during implantation. In contrast, larger granules provide increased mechanical stability but have lower surface area-to-volume ratio, which may result in slower integration with the host bone. Granules ranging from 0.1 to 5 mm in diameter [34] are commonly used in both orthopedic and dental applications, as this range offers a good balance between handling ease, effective packing within bone defects, and a favorable biological response.

This size requirement presents a challenge for mass-producing 3DP HA/PCL granules, as they tend to agglomerate during the infiltration process. Unlike larger samples that can be handled individually, small granules often bind together during solidification and solvent leaching, making it difficult to maintain their individual form. In this study, we address this issue by developing 3DP HA/PCL bone graft granules using liquid agitation to prevent agglomeration. We investigate the effects of varying PCL solution concentrations and different agitating liquids on the microstructural, mechanical, and biological properties of the resulting composites. Our goal is to identify fabrication parameters that achieve 3DP HA/PCL granules with enhanced mechanical properties while retaining their granular form and biocompatibility, essential for potentially used in bone grafting.

## 2. Materials and Methods

### 2.1. 3DP HA Fabrication

**Figure 1:**
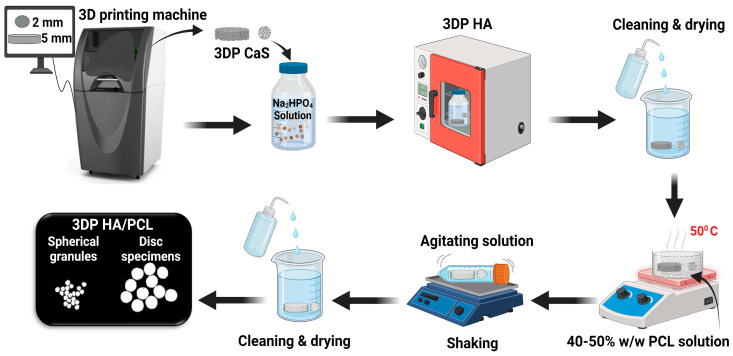
The fabrication process of 3DP HA/PCL: 3DP HA was prepared by using binder jetting 3D printing combined with a phase transformation process and then PCL solution infiltration in combination with liquid agitation.

Three-dimensionally printed hydroxyapatite (3DP HA) was fabricated by a combination of binder jetting and low-temperature phase transformation (Figure 1). A graphical 3D image of the samples including spherical granules with 2.0 mm in diameter (for XRD, SEM, FTIR and compression load) and a disc with 5 mm in diameter and 1 mm thick (for TGA, contact angle, diametral tensile strength and cell proliferation) were designed and imported into the binder jetting three-dimensional printing machine (Projet160, 3D Systems, Rock Hill, SC, USA) to print the samples using calcium sulfate-based powder (Visijet PXL Core, 3D Systems, Rock Hill, SC, USA) and a liquid binder (Visijet PXL Clear, 3D Systems, Rock Hill, SC, USA). After printing, the fabricated calcium sulfate samples were immersed in 1M disodium hydrogen phosphate solution (Sigma Aldrich, St. Louis, MI, USA) at 100 °C in the air-circulated oven for 48 h to convert the as-printed calcium sulfate to hydroxyapatite by ion exchange reaction, as described in detail elsewhere [23]. After that, the obtained 3DP HA samples were cleaned with distilled water and oven-dried. The PCL sheet used for FTIR and contact angle analysis was prepared by solution casting of the PCL solution into a petri dish, followed by solidification in deionized water.

### 2.2. PCL Infiltration

Three-dimensionally printed HA/PCL was prepared by the PCL solution infiltration in combination with liquid agitation (Figure 1). Firstly, polycaprolactone, M_n_~10,000 (Sigma Aldrich, USA), was dissolved in N-Methyl-2-Pyrrolidone (NMP, TSquare Synergy (Thailand) Co., Ltd., Bangkok, Thailand) to achieve a concentration of 40% and 50% *w*/*w*. Three-dimensionally printed HA was then solution-infiltrated by placing it in PCL solution at 50 °C for 15 min. The infiltrated samples were then transferred into a bottle that contained one of the agitating liquids, including deionized water (DI), NMP, and a mixture of NMP/DI = 90:10 *wt*/*wt*. The bottle was then agitated continuously for 45 s to prevent the agglomeration of the samples. The samples were taken out, cleaned in deionized water for 24 h, and dried at room temperature for 48 h. Table 1 shows the infiltration conditions used for fabricating 3DP HA/PCL.

### 2.3. Physical and Mechanical Properties

#### 2.3.1. Viscosity 

The viscosity measurement of the PCL solutions was carried out using a controlled strain rheometer (ARES-G2, TA Instruments, New Castle, DE, USA), operated at a temperature of 25 °C using Bob and Cup stainless steel (Bob diameter 25 mm, Bob length 42 mm and Cup diameter 30 mm). Frequency sweep tests were carried out with a shear rate set in the range of 1 to 500 s^−1^ for the rotational test. 

#### 2.3.2. X-ray Diffraction 

Phase composition of the samples was evaluated by using an X-ray diffractometer (XRD, Rigaku TTRAX III, The Woodlands, TX, USA) with CuKα source (Kα = 0.15406 nm) operating at 300 mA and 50 kV. XRD measurement was conducted at 15–40° 2Ɵ, a scan speed of 3° min^−1^, and a step angle of 0.02°. The XRD spectra were analyzed using JADE software version 9.7, which allowed searching for ICDD database products. 

#### 2.3.3. Fourier-Transform Infrared Spectroscopy

Three-dimensionally printed HA or 3DP HA/PCL was ground together with potassium bromide (KBr) and a KBR pellet was formed by a compression die. The samples were then examined using an FT-IR Spectrometer (Spectrum One, PerkinElmer, Shelton, CT, USA) at the wavenumber 4000–400 cm^−1^ with a resolution of 4 cm^−1^ using a TGS detector. The PCL sheet was examined by FT-IR Imaging Microscope (Spectrum Spotlight 300, PerkinElmer, USA) at the wavenumber 4000–600 cm^−1^ using the micro-attenuated total reflectance (Micro-ATR, PerkinElmer, Shelton, CT, USA) technique at the wavenumber 4000–600 cm^−1^ with a resolution of 4 cm^−1^ using an MCT detector.

#### 2.3.4. Thermogravimetric Analysis

PCL content in the sample was determined by thermogravimetric analysis (TGA) using an STA analyzer (STA 449C, NETZSCH Instruments North America, LLC, Burlington, MA, USA) by heating the sample in an alumina crucible from 25 to 800 °C in an air atmosphere at a heating rate of 10 °C min^−1^. Infiltration content was determined as the weight loss percentage of PCL after pyrolysis in the TGA thermogram.

#### 2.3.5. Contact Angle 

Surface wettability was measured by a contact angle technique using a goniometer (Model 200, Rame-Hart Instrument Co., Succasunna, NJ, USA) and deionized water was employed as a dropping liquid. 

#### 2.3.6. Mechanical Properties

Compression load resistance of the spherical granules and diametral tensile strength (DTS) of the disc specimens were performed on a universal testing machine (Dynamic, AGX-100kNV, Shimadzu, Kyoto, Japan) by similarly compressing the samples between two metallic platens at a constant crosshead speed of 1.0 mm min^−1^. The maximum load resistance was then determined. In the case of DTS, the compressive load was applied in a diametral plane, which is perpendicular to the longitudinal axis. DTS value was calculated using the equation DTS = 2L/πDh, where L is the maximum load, D the diameter, and h the thickness of the specimen. All the tests were carried out at 23 °C and 50% RH. 

#### 2.3.7. Microstructures 

Surface and core microstructures of the sample were carried out using a scanning electron microscope (SEM, JEOL JSM-7800F Prime, Akishima, Japan) at an accelerating voltage of 1 kV. Prior to SEM observation, samples were dried at 80 °C for 24 h and gold-sputtered under a vacuum to prevent overcharging and improve conductivity.

### 2.4. In Vitro MC3T3-E1 Cell Proliferation

The samples were soaked in α-MEM completed medium (Gibco, Thermo Fisher Scientific, Waltham, MA, USA) overnight and placed in a 48-well plate. MC3T3-E1 pre-osteoblast cells (ATCC CRL-2593, RRID:CVCL_5440, ATCC, Manassas, VA, USA) were seeded on the surface at a concentration of 5 × 10^5^ cells/mL by adding 100 microliters per well (resulting in 5 × 10^4^ cells per sample). The samples were cultured in a CO_2_ incubator at 37 °C, 5% CO_2_, and 95% RH. The proliferation of pre-osteoblast cells on the samples was measured using the Alamar blue assay (Invitrogen, Thermo Fisher Scientific, USA) at 1, 3, 7, and 14 days. The culture medium was removed, and samples were washed three times with Dulbecco’s Phosphate-Buffered Saline (Gibco, Thermo Fisher Scientific, USA). Alamar blue solution (20 microliters) was added to each sample containing 200 microliters of culture medium. The plates were incubated in the CO_2_ incubator for 2 h. Absorbance was measured at 570/600 nanometers using a microplate reader (ASYS UVM340, Biochrom Ltd., Cambridge, UK), and optical density (OD) was obtained. The control samples including the cell control (well with cells, but no sample) and blank control (well with no cells) were also cultured at each time to ensure the reliability of the results. In addition, 3DP HA was also employed as the control sample for result comparison. The morphology of cells was determined by fixing the cells using glutaraldehyde solution (25% EM-grade, Electron Microscopy Sciences, Hatfield, PA, USA) for 2 h at room temperature, dehydrated by series of ethanol (RCI Labscan Co., Ltd., Bangkok, Thailand) followed by critical point drying (CPD300, Leica Microsystems, Buffalo Grove, IL, USA) using liquid CO_2_. The samples were gold-sputtered before observation by a scanning electron microscope (JEOL JSM-5410, Japan).

### 2.5. Statistical Analysis

Results are presented as mean ± standard deviation (SD). Given the normal distribution of data, the significant differences in properties amongst samples were analyzed using a one-way analysis of variance (ANOVA) and Bonferroni post-hoc testing. A value of *p* < 0.05 was considered significant.

## 3. Results

**Figure 2:**
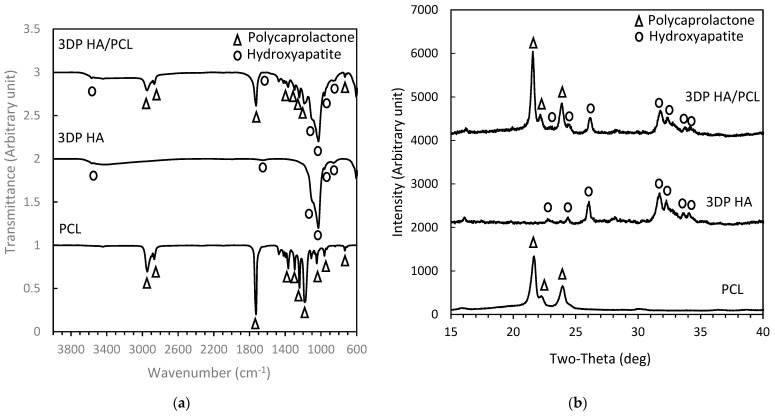
XRD patterns (**a**) and FTIR spectra (**b**) of 3DP HA/PCL, 3DP HA, and polycaprolactone.

Table 2 shows the measured shear viscosities of the 40% and 50% PCL solutions used for the solution infiltration process. The 50% PCL solution exhibited significantly greater viscosity compared to the 40% solution. From the processing steps employed, 3DP HA/PCL granules were fabricated successfully without agglomeration. Figure 2a displays the representative FTIR spectrum of the 3DP HA/PCL. The samples displayed main spectra bands of HA at 1029–1126 cm^−1^ corresponding to the ν_3_ mode of PO_4_^3−^, HPO_4_^2−^ bands at 858 cm^–1^, ν_1_ mode of PO_4_^3−^ band at 962 cm^−1^, OH^−^ bands at 3700–2400 cm^−1^, and ν_2_ mode of H_2_O band at 1658 cm^–1^ and of PCL at 1728 cm^−1^, which corresponded to carboxyl stretching, 2946 cm^−1^(asymmetric CH_2_ stretching), 2867 cm^−1^ (symmetric CH_2_ stretching), 1294 cm^−1^ (C–O and C–C stretching in the crystalline phase), 1179 cm^−1^, and 1241 cm^−1^ (symmetric and asymmetric C-O-C stretching) [36]. 

Figure 2b displays the representative XRD patterns of the 3DP HA/PCL after infiltration. The XRD peaks for both 3DP HA and PCL samples are broad, indicating their low crystallinity. The peaks were indexed to the characteristic patterns of hydroxyapatite at 2-theta values of 22.7, 24.4, 25.9, 31.6, 32.3, 33.5, and 34.1, and of PCL at 2-theta values of 21.6, 22.2, and 23.9 [37]. The FTIR and XRD analysis demonstrate the coexistence of both HA and PCL within the fabricated composite, validating the effectiveness of the fabrication process. No alteration in the peaks or phase compositions of the samples due to the infiltration conditions was observed.

Table 3 shows the contact angles of 3DP HA/PCL, 3DP HA, and PCL. The use of NMP and NMP-DI resulted in a contact angle of zero, similar to that of 3DP HA, while using DI led to a significantly higher contact angle, comparable to that of PCL. For DI, the 50% PCL solution exhibited a statistically greater contact angle compared to the 40% PCL solution. This indicates that using DI decreased the wettability of the samples.

**Figure 3:**
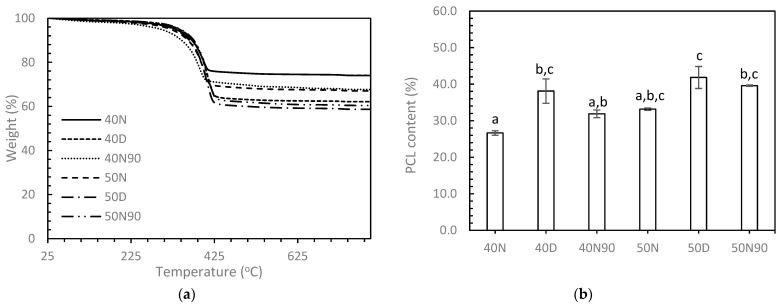
PCL content in 3DP HA/PCL as determined by TGA analysis (n = 3). (**a**) TGA thermograms; (**b**) PCL infiltration content. Means that do not share a symbol (a, b, and c) are significantly different.

Figure 3 presents a comparison of the PCL content in 3DP HA/PCL composites resulting from infiltration using various PCL solution concentrations and agitating liquids. For the same agitating solution, the 50% PCL solution tended to result in greater PCL content compared to the 40% PCL solution; however, no statistically significant difference was observed between samples using different PCL solution concentrations. When considering the same PCL solution concentration, NMP resulted in the lowest PCL content, while DI produced the highest PCL content. Using a mixture of DI and NMP yielded intermediate PCL content. However, a significant difference in PCL content was observed between samples 40N and 40D when using the 40% PCL solution only.

**Figure 4:**
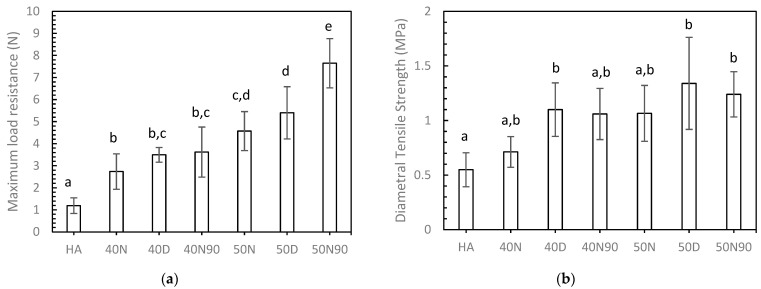
Mechanical properties of 3DP HA/PCL: (**a**) maximum compression load (n = 10); (**b**) diametral tensile strength (n = 5). For each property, means that do not share a symbol (a, b, c, d, and e) are significantly different.

Figure 4 shows the mechanical properties of 3DP HA/PCL compared to those of 3DP HA alone. For compression load resistance, all 3DP HA/PCL samples demonstrated significantly higher values than the 3DP HA, with the extent of improvement varying based on the infiltration conditions. Specifically, the 50% PCL solution produced significantly higher compression load resistance compared to the 40% PCL solution for the same agitating solution. When comparing the same PCL solution concentration, a mixture of DI and NMP resulted in intermediate compression load resistance. Notably, significant differences in compression load resistance were observed between 50N or 50D and 50N90 when using the 50% PCL solution. For DTS test, only the 40D, 50D, and 50N90 samples showed significantly higher values than the 3DP HA. Although the 50% PCL solution tended to yield higher DTS than the 40% solution for the same agitating solution, no statistically significant differences were observed between the different PCL solution concentrations. Among the same PCL solution concentrations, NMP resulted in the lowest DTS, DI in the highest DTS, and a mixture of DI and NMP produced intermediate DTS. However, no significant differences in DTS were seen among the different agitating liquids. Overall, among all samples, 50N90 exhibited the greatest compression load resistance, while both 50D and 50N90 showed the highest DTS values.

**Figure 5:**
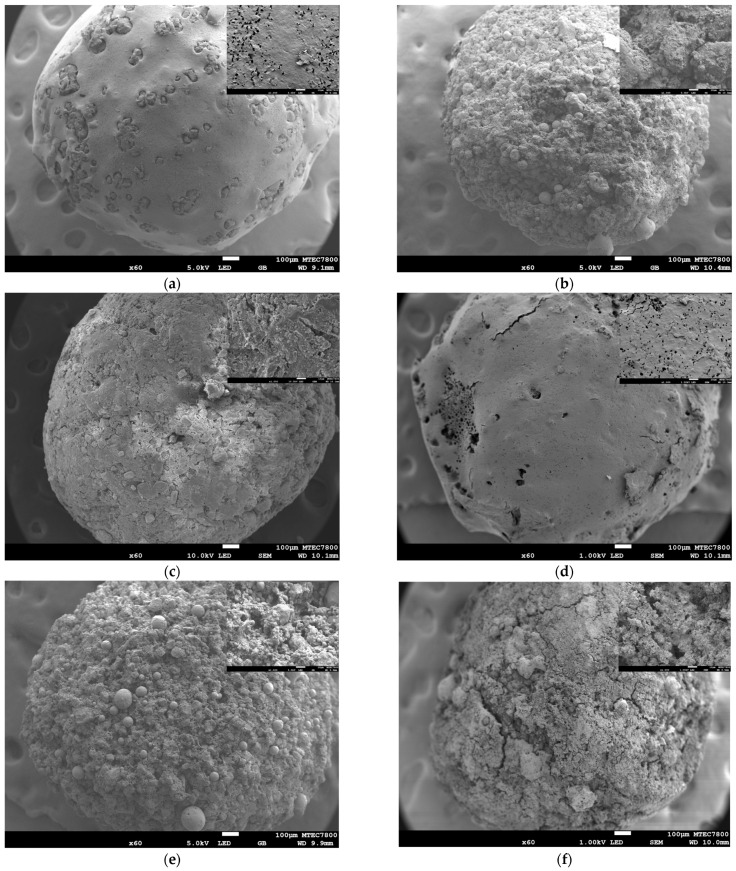
SEM images showing the surface microstructure of 3DP HA/PCL granules: (**a**) 40D; (**b**) 40N; (**c**) 40N90; (**d**) 50D; (**e**) 50N; (**f**) 50N90. Magnification ×60, scale bar= 100 μm. Inset images show the enlarged details at a magnification of ×1000, scale bar = 10 µm.

**Figure 6:**
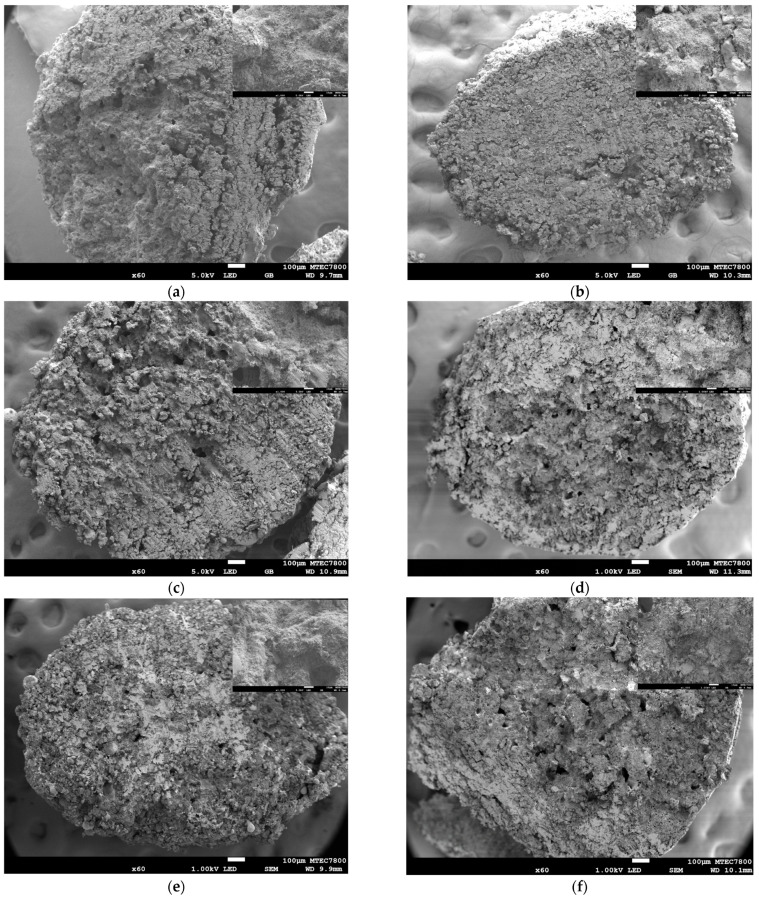
SEM images showing the core microstructure of 3DP HA/PCL granules: (**a**) 40D; (**b**) 40N; (**c**) 40N90; (**d**) 50D; (**e**) 50N; (**f**) 50N90. Magnification ×60, scale bar= 100 μm. Inset images show the enlarged details at a magnification of ×1000, scale bar = 10 µm.

The microstructure of the final construct is crucial, as it significantly influences the properties of the composite, particularly in determining whether the PCL penetrates into the internal core or remains on the surface of the 3DP HA. Figure 5 illustrates the surface microstructures of samples infiltrated with varying PCL solution concentrations and different agitating liquids. Regardless of the PCL solution concentration, the use of DI water as an agitating liquid similarly resulted in a relatively dense and smooth PCL coating with minute pores on the granule surface. This effect was due to the instant solidification of the infiltrated PCL solution on the granule surfaces upon contact with DI water, which acts as a non-solvent for PCL. Conversely, the use of NMP resulted in a porous microstructure, characterized by the infiltration of PCL into the initial pores of the 3DP HA and the distribution of phase-separated, globular-shaped PCL throughout the surface, which left the HA crystals prominently exposed. This was due to the diffusion of the initially infiltrated PCL solution out of the granule upon contact with the same solvent, NMP, in the agitating liquid. Utilizing an NMP-DI mixture produced an intermediate microstructure, marked by an irregular and discontinuous PCL coating. This coating was less dense compared to that achieved with DI alone and exhibited a porous structure due to PCL infiltration within the initial pores of the 3DP HA, similar to the structure observed when using NMP alone. Figure 6 shows the core microstructures of samples. Unlike the surface microstructure, no distinct differences among infiltration conditions were observed. All samples similarly exhibited the porous core microstructure resulting from the infiltration of PCL into the initial pores of the 3DP HA.

**Figure 7:**
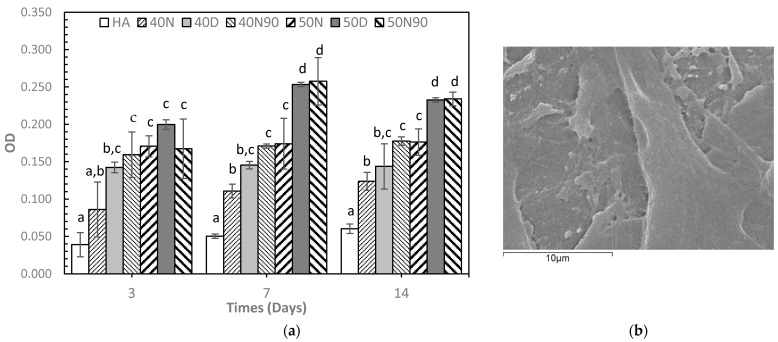
MC3T3-E1 cell proliferation by alamar blue assay: (**a**) Optical density at cultured times of 3, 7, and 14 days (n = 3). For each time, means that do not share a symbol (a, b, c, and d) are significantly different. (**b**) Representative SEM image of cell morphology growth on the 50N90 sample at 3 days, ×5000.

Figure 7a shows the proliferation of MC3T3-E1 pre-osteoblast cells on the 3DP HA/PCL samples using various infiltration conditions compared to those of 3DP HA over different culturing periods. The results indicate that cells can grow on all samples, as evidenced by an increasing trend in optical density (OD) over time. At each culturing period, cell proliferation on all 3DP HA/PCL samples was significantly greater than that on 3DP HA alone, except for 40N on day 3. For the same agitating solution, samples using the 50% PCL solution resulted in significantly greater OD values compared to the 40% PCL solution, except on day 3, when the OD values between different solution concentrations did not reach a statistical difference. When considering the same PCL solution concentration, NMP resulted in the lowest OD values, while DI and a mixture of NMP and DI produced significantly higher OD values, especially at longer culture times (7 and 14 days) and with the higher solution concentration (50%). Overall, among all samples, 50D and 50N90 consistently exhibited the greatest OD values at each cultured period. Figure 7b illustrates the surface of the samples after culturing MC3T3-E1 pre-osteoblast cells at 3 days, showing the typical morphology that the cells can grow and spread to adhere to the surface of sample.

## 4. Discussion

In general, the production of bone graft granules involves milling, where materials are first synthesized into a bulk form, ground into granules, and then sieved into desired size ranges [38,39,40,41]. However, these methods can result in irregularly shaped granules with a wide size distribution, which may affect the consistency of clinical outcomes. Advancements in 3D printing and additive manufacturing like binder jetting as described in this study have revolutionized the production of bone graft granules, allowing for changes to and control over the size and shape of the granules, leading to more uniform and reproducible products. The infiltration of PCL into 3DP HA granules followed by immediately agitating in liquid has been shown to be a viable process to produce 3DP HA/PCL granules without agglomeration or binding among infiltrated granules. The agitation of infiltrated granules causes the motion of granules, causing them to be separated from each other while simultaneously partially solidifying the infiltrated PCL, rendering them less capable of binding together, resulting in the separation of the infiltrated granules before subsequently going through full solidification and solvent leaching out in the water. The granules fabricated in this study were spherical, with a diameter of 2 mm, falling within the typical size range of 0.1 mm to 5 mm commonly used for bone grafting [34]. The size and shape can also potentially be adjusted as needed for specific applications.

The concentration of PCL solution and types of agitating liquids were seen to affect the characteristics and properties of the resulting composite granules in various ways depending on the properties concerned. Regarding reinforcement, a higher content of infiltrated PCL enhances the mechanical properties of the 3DP HA/PCL composite. The infiltration process allows for PCL to penetrate and fill the pores within the 3DP HA matrix, creating a more homogeneous and dense structure. This reduces internal voids, improves load distribution, and dissipates energy under mechanical stress. The increase in concentration PCL solution is theoretically desirable, as it would increase the content of PCL in the composite, but high concentration also increases the viscosity of the solution, making it more difficult to penetrate into the preformed structure. In this study, two concentrations were employed, 40% and 50%, which were found from a preliminary study, and previous study [33] stating that a higher concentration yielded high viscosity, which was not suitable for solution infiltration. The agitating liquids studied, chosen based on their solvating ability, included DI, NMP, and a mixture of these liquids. DI was selected since it is a non-solvent liquid for PCL and is typically used as cleansing media after solution infiltration. NMP is a colorless water-miscible super-solvent with a high boiling point, low viscosity, low toxicity, and good biocompatibility that is frequently used to dissolve a polymer–drug solution along with other additives or biomedical polymer preparations [42,43] and was also employed as a solvent of PCL in this study. The mixture of DI and NMP was employed to combine the non-solvent and solvent properties in a single liquid. 

When analyzing the properties of 3DP HA/PCL as a result of the infiltration process, the effect of both solution concentration and agitating liquids have to be considered together. The final content of PCL in 3DP HA/PCL was not affected by the concentration of PCL solution or the agitating liquids employed. This was probably due to the counteracting effect of the increase in PCL content in the solution and the decrease in penetration ability due to the increase in viscosity. Although the infiltrated amount of the lower viscosity 40% solution might be greater than that of the higher 50% due to its lower viscosity, the PCL content in the 40% solution is lower. Types of agitating liquid had more effect on PCL content since its function is to partially solidify the infiltrated samples and retain the PCL within the composite structure. Using NMP was considered to be the least effective since it was the same as the solvent in the PCL solution, while DI would be the most effective for PCL since it is a non-solvent. NMP-DI mixture would possess an intermediate effect. However, this was thought to be confined to the contact front at the surface of the samples only due to short period of the process. Accordingly, the observed microstructure of 3DP HA/PCL, particularly the surface microstructure, changed with infiltration conditions, producing either a dense coating, irregular and discontinuous coating, or porous and phase-separated morphology depending on the agitating liquid. These changes in microstructure corresponded closely with the wettability of 3DP HA/PCL, as evidenced by contact angle measurements. The contact angle of 3DP HA/PCL increased from zero to a value closer to that of PCL as the microstructure transitioned from a porous morphology to a dense coating and as the concentration of PCL solution increased. Therefore, although the final PCL content of all infiltration conditions was comparatively similar, the characteristics of the infiltration were dissimilar and would affect the properties of 3DP HA/PCL.

**Figure 8:**
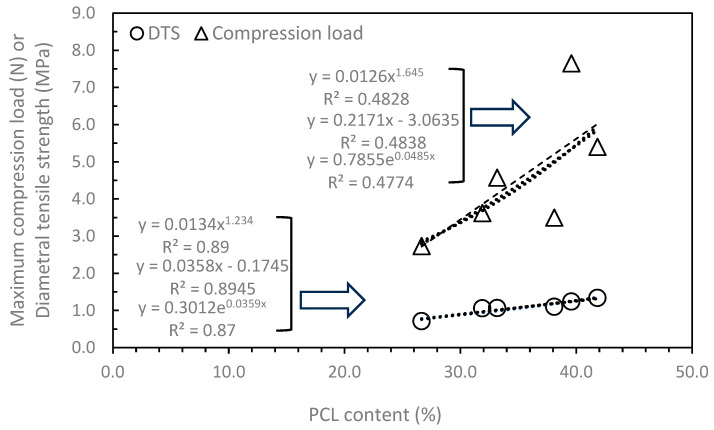
The correlation between the infiltrated PCL content and the compression load or diametral tensile strength (DTS). The dotted and dashed lines display the trendline of the data generated by regression curve fitting, along with the fitting equations.

The 3DP HA/PCL samples exhibited significantly higher mechanical properties compared to the 3DP HA samples, demonstrating the effectiveness of the infiltration process as intended. The greatest compressive load resistance of the 3DP HA/PCL granules (50N90) was 7.65 N. If the cross-sectional area at the largest diametral area was considered, the compressive strength of the granule was approximately 2.4 MPa, which is about seven times greater than that of 3DP HA granules. The compressive strength of cortical bone was reported to range from 100 to 230 MPa, while the compressive strength and tensile strength of trabecular bone has been reported to lie between 0.1 and 14 MPa and 1.3 and 3.5 MPa. [44,45]. The mechanical properties of 3DP HA/PCL granules are thus comparable to those of trabecular bone. The extent of improvement varied based on the infiltration conditions. While the infiltrated PCL content was not significantly affected by these conditions, the mechanical properties of 3DP HA/PCL varied with different solution concentrations and agitating liquids. The maximum compression load resistance of individual granules increased in the following order: 40N < 40D, 40N90 < 50N < 50D < 50N90. This indicates that using a low PCL solution concentration and NMP resulted in the lowest compression load resistance, whereas higher PCL solution concentrations and a DI or NMP-DI mixture provided higher load resistance. Similar trends were observed in the DTS test, but the degree of influence was less pronounced, resulting in no statistically significant differences between the different PCL solution concentrations and agitating liquids. These differences can be attributed to the distinct modes of mechanical response in both tests and the microstructure of the 3DP HA/PCL produced under different infiltration conditions. In the compression test, the load was applied uniaxially on the surface of the sample. The observed surface microstructure of a dense PCL coating when using DI or an irregular and discontinuous PCL coating when using an NMP-DI mixture helped resist the compression load better than a porous surface microstructure with phase-separated and globular-shaped PCL when using NMP. In the DTS test, the tensile load was produced perpendicularly to the applied compression load. Therefore, the influence of surface microstructure was less significant in this case, with the core microstructure being the main influencing factor. Consequently, less influence of solution concentration and agitating liquid on DTS was observed. Generally, the mechanical properties of porous materials increase with density, which can be described by empirical or semi-analytical models using linear, exponential, or power functions [46,47,48]. When replotting PCL content, which corresponds to the density of 3DP HA/PCL as a function of compression load or DTS, as shown in Figure 8, it can be seen that the DTS was well represented by least-squares curve fitting for the above-correlated functions, R^2^ ≈ 0.87–0.89. In contrast, poor fittings of the compression load were noted, R^2^ ≈ 0.48. Therefore, it indicates that the underlying controlling mechanisms differ between compression load resistance and DTS, as discussed earlier.

Although PCL infiltration enhances the mechanical properties of 3D-printed HA, its biocompatibility must also be considered for potentially use as a synthetic bone graft. Preliminary alamar blue assays were performed to evaluate and compare the ability of 3DP/HA to support cell proliferation on the surface among samples. Cell interactions are influenced by the microstructure, macrostructure, surface chemistry, and surface topology of the materials [49,50]. The use of alamar blue in our study allowed for us to compare the biocompatibility of different conditions by directly measuring the proliferation rates of pre-osteoblast cells (MC3T3-E1) in contact with the various composite materials. The rationale for using alamar blue lies in its sensitivity and ability to provide continuous, real-time data on cell proliferation. This makes it particularly useful for comparing the biocompatibility of materials, as it directly reflects how well cells are able to grow on the composite under different experimental conditions. In this study, we observed that changes in the microstructure and mechanical properties of infiltrated samples, produced under different infiltration conditions, affected pre-osteoblast interactions. Specifically, increased cell proliferation followed trends similar to the increases in mechanical properties, particularly compression load resistance. Previous studies have shown that changes in the mechanical environment, such as substrate stiffness, influence cell adhesion, proliferation, and differentiation [51]. The changes in stiffness and microstructure induced by the infiltration process may create an optimal environment in terms of mechanosensing, hydrophilicity, and architecture to support cellular growth. Stiffer substrates have been reported to enhance cell functions, including attachment, spreading, proliferation, differentiation, and gene expression [52]. Additionally, the bioactive exposed HA crystals on the surface of the composite, which support osteoblast proliferation and differentiation, should also be considered. However, the exact underlying mechanisms are not confirmed and are beyond the scope of this study. Future research should be conducted to elucidate these mechanisms. 

A limitation of this study is the absence of additional assays, such as ALP (alkaline phosphatase) activity and other in vitro bioactivity tests, which are critical for assessing cell differentiation and early markers of osteogenic potential. These assays are essential for understanding the material’s ability to support not only cell proliferation but also the maturation of bone-forming cells and the deposition of calcium. The inclusion of such tests would provide a more comprehensive evaluation of the material’s bioactivity. Future research will address these limitations, incorporating ALP assays, calcium production tests, and other bioactivity markers to thoroughly assess osteogenic potential. Additionally, animal studies and clinical investigations will be conducted to validate the safety, long-term effectiveness, and integration of the material in bone regeneration applications. These studies will be crucial in determining the material’s suitability as a bone graft for clinical use.

## 5. Conclusions

Within the scope of this study, using a 50% PCL solution with the NMP-DI mixture as an agitating liquid demonstrated the desired microstructural, mechanical, and biological properties for 3DP HA/PCL granule fabrication. However, further evaluation of its bioactivity, including cell differentiation assays such as ALP activity and calcium production, along with comprehensive safety and effectiveness assessments in animal models and clinical investigations, should be performed to fully validate its potential use as a bone graft in bone regeneration

## Figures and Tables

**Table 1:** Infiltration conditions for fabricating 3DP HA/PCL.

Samples	PCL Solution Concentration (% *wt*/*wt*)	Agitating Liquid
40D	40	DI
40N	40	NMP
40N90	40	NMP:DI = 90:10
50D	50	DI
50N	50	NMP
50N90	50	NMP:DI = 90:10

**Table 2:** Shear viscosity of PCL solution used for infiltration as measured by a rotational rheometer (error bars = standard deviation, n = 3).

Samples	Shear Viscosity (Pa·s)
40% PCL solution	0.48 ± 0.02
50% PCL solution	0.79 ± 0.03

**Table 3:** Contact angle of 3DP HA/PCL (Mean ± SD, n = 3). Means that do not share a symbol (a, b) are significantly different.

Samples	Contact Angle (Degree)
40D	26.3 ± 0.71 ^a^
40N	0 ± 0.00
40N90	0 ± 0.00
50D	69.8 ± 5.11 ^b^
50N	0 ± 0.00
50N90	0 ± 0.00
PCL	69.3 ± 3.26 ^b^
3DP HA	0 ± 0.00

## Data Availability

The original contributions presented in the study are included in the article; further inquiries can be directed to the corresponding author.
